# Co-Designing a Web-Based Decision Aid Tool for Employees Disclosure of Mental Health Conditions: A Participatory Study Design Using Employee and Organizational Preferences

**DOI:** 10.2196/23337

**Published:** 2020-11-06

**Authors:** Elizabeth Stratton, Isabella Choi, Dorian Peters, Rafael A Calvo, Samuel B Harvey, Nicholas Glozier

**Affiliations:** 1 Central Clinical School Faculty of Medicine and Health University of Sydney Sydney Australia; 2 Dyson School of Design Engineering Imperial College London United Kingdom; 3 School of Electrical and Information Engineering The University of Sydney Sydney Australia; 4 School of Psychiatry University of New South Wales Sydney Australia; 5 Black Dog Institute Sydney Australia; 6 St George Hospital Sydney Australia

**Keywords:** employee disclosure, decision aid tool, mental health, web-based

## Abstract

**Background:**

Decisions of whether to disclose mental health conditions are extremely personal and require the consideration of multiple factors associated with the disclosure process (eg, weighing the risks and benefits). Decision aid tools help people make these complex decisions. Such an aid needs to be confidential, easily accessible, and easy to use with the potential to access the tool on multiple occasions. Web programs are well suited to meet these requirements and, if properly developed, can provide feasible, accessible, affordable, and effective workplace interventions.

**Objective:**

This study aims to gain insights from potential end users, in this case both employees and organizations, into what type of components including language, style, and content would avoid potential stigma and ensure that elements of clear value for users would be built into a web-based decision aid tool that aims to assist employees in making decisions about the disclosure of their mental health condition at work.

**Methods:**

A participatory design approach was used to allow developers, researchers, experts, and end users to collaborate in co-designing the tool. During the user research phase of the development of the web-based tool, a participatory design workshop approach was selected as a part of a larger study of focus groups. Australian employees and managers in rural, suburban, and urban locations participated in an exploratory qualitative study involving participatory workshops designed to elicit their perspectives and preferences for a decision aid tool.

**Results:**

A total of 2 workshops were conducted with 13 participants. The majority were from a transport company (9/13, 69%), male (8/13, 62%), and employed full time (11/13, 85%). Six employees had previous experience disclosing their own mental health condition, and 7 were in a supervisory role and had previously been disclosed to. In any co-design development, there are certain trade-offs that need to be made between the views of experts, developers, end users, and the available budget. In this specific instance of a very delicate, personal decision, the end users provided valuable design insights into key areas such as language, and they were very antipathetic to a key feature, the avatar, which was thought to be desirable by experts and developers. Findings including aspects of the tool where all stakeholders were in agreement, aspects where some stakeholders disagreed and adaptations were implemented, where disagreements could not be implemented because of financial constraints, and misalignment between stakeholders and how to decide on a balance were shared.

**Conclusions:**

The co-design with a lived experience approach is useful for contributing much to the design, language, and features. The key in this study was balancing the needs of the workers and the potential impact for the managers and organizations, while ensuring legislation and regulation requirements were upheld.

## Introduction

Disclosure of a mental health condition is a necessary first step to help seeking in the workplace [1] as employers are not legally obliged to provide individual support before disclosure [2]. A lack of disclosure limits opportunities to access workplace support that could allow employees to maintain performance in their role or successfully return to work [3]. Once a condition is disclosed, employees may be theoretically protected under the relevant legislation and antidiscrimination laws.

For people in employment, deciding to disclose a mental health condition is often complex and requires thoughtful decision making to ensure personal, legal, and employment risks are best managed. The majority of employees with a mental health condition favor nondisclosure as their preferred option [3]. A global survey of employees with major depressive disorder showed that 71% preferred to conceal their condition from others in the workplace and almost half of those (47%) feared they would lose their job if they disclosed [4].

Decisions on whether to disclose are highly personal and require the consideration of multiple factors associated with a disclosure process, for example, weighing the risks and benefits [5]. The capacity to make decisions may be more complex in this population as those with depression and anxiety show impaired decision-making behavior [6,7]. While research does not yet provide clear-cut guidelines for disclosure, facilitators and barriers involved in the disclosure decision-making process in workplaces have been evaluated [8]. The decision to disclose mental ill health according to those who had disclosed in the past and those who, as managers, had received disclosures appears to be determined primarily by consideration of barriers to disclosure and fear of negative consequences rather than any perceived facilitators or benefits [9]. Despite recent progress toward increasing knowledge of public stigma and discrimination [10], they remain to be a significant issue. Discrimination toward those with a mental health condition is a common problem in the workplace globally [11].

In addition to the consequences of disclosing, there are a number of components in the disclosure decision-making process. Employees must decide *who* the best person to disclose to is, for example, a supervisor or manager*, what* information about their condition they will disclose, and *when* to do so. These decisions rest solely with the employee and/or their close supports and clinicians, but many have little help in the decision-making process.

Decision aid tools offer a means to help people manage these complexities. Such tools have commonly been used in decision making regarding medical treatment options [12]. A systematic review showed that decision aids for people facing treatment decisions produced less decisional conflict and led them to have a more active role in the decision-making process [13]. In an organizational mental health context, a paper-based decision aid tool for people with a severe mental illness in secondary care services reduced disclosure decisional conflict and improved individual empowerment when seeking employment [14]. This tool, CORAL (Conceal or ReveAL), developed and evaluated by Henderson et al [14] was the first intervention to support decision making about disclosure in the employment context. In collaboration with Henderson [14] from the CORAL study, we developed a decision aid tool READY (Reducing dEcisionAl conflict, a Decision aid tool for emploYees). The labels of the original modules in CORAL were used to form a framework for READY. The authors developed the content of the tool based around (1) relevant legislation and regulation specific to Australia, (2) delivery to those with any mental health condition when they are still at work, and (3) language for low mental health literacy. The prototype was then presented to a larger expert group of clinicians, researchers, mental health professionals, peer support workers, and work health and safety and vocational officers.

When designing a disclosure decision aid, a tool needs to incorporate counterfactual and potentially positive aspects that, from our focus groups, do not appear to be considered in decisions about disclosing mental health issues in the workplace [9]. Such an aid needs to be confidential, easily accessible, and easy to use, with the potential to access the tool on multiple occasions. Web programs are well suited to meet these requirements and, if developed properly, can provide feasible, accessible, affordable, and effective workplace interventions [15,16]. In addition, they provide around-the-clock access [17] as employees might consider their options outside of their normal work hours.

As with all digital interventions, a tool can only be effective if it engages its intended audience [18], which means that considering end users’ needs and preferences during the design process is crucial. Participatory design methods allow researchers to co-design interventions with potential end users by eliciting user perspectives, preferences, and ideas [19]. This approach is used to ensure that these interventions are more likely to be engaging and effective for the intended audience [19-21].

In this study, results are presented from the user research phase (co-design) of the development of a web-based tool that aims to assist employees in making decisions about the disclosure of their mental health condition at work. A participatory design workshop approach was selected. The aim of this study is to gain insights from potential end users into what type of components, including language, style, and content, would avoid potential stigma and to ensure that elements of clear value for users would be built into the decision aid tool to fundamentally increase uptake and ongoing engagement.

## Methods

### Study Setting

Participatory workshops were conducted as part of a large Australian-based research collaboration (Well@work) with 15 industry partners, all of whom were invited at an annual meeting to participate; 2 industry partners agreed to participate.

### Sample/Recruitment

Participants were recruited from a First Responder Association and a transport company in New South Wales, Australia. Recruitment was conducted via internal organizations inviting those employees who had already formally disclosed their mental health condition and any supervisors who had received disclosures. Within these organizations, 23 employees and supervisors were selected by the human resources team within their organization and were invited through an email sent out via their workplace administration to attend the workshops. The research team had no contact with the participants until the day the study was conducted. Ethical approval was obtained from the University of Sydney Human Research Ethics Committee (project no. 2016/766).

Participants were identified as belonging to 1 of 2 categories: (1) those who had disclosed their mental health conditions in the workplace, that is, *disclosure group* and (2) those who supervised employees within their organization and therefore received disclosures, that is, *authority group*.

### Procedures

After obtaining written informed consent, each participant was asked about their role within their organization and whether or not they wished to disclose their mental illness as part of the workshops. Overall, 2 activity-based workshops were conducted in July and August 2017.

The facilitator (ES) was guided by a user experience specialist (DP), an experienced workplace qualitative researcher. The facilitator (ES), author (DP), and the principal investigator (NG) constructed the discussion guide (Multimedia Appendix 1) using semistructured topic guides and activities. An activity-based participatory design approach was adopted for the workshops with flexibility to ensure that the facilitator could follow-up on any important remarks or seek clarification of understanding. This qualitative strategy allowed access to a variety of perspectives and experiences and maximized the ability to develop an effective, appropriate intervention by being able to open dialogue and expand on responses when clarification was needed.

### Prototype Development

A prototype was developed based on previous literature on a paper-based decision aid tool [14] and in consultation with a group of workplace disclosure experts. The experts assisted in defining the core concepts, requirements, and features to adapt the tool. The expert group consisted of clinicians, researchers, mental health professionals, peer support workers, and work health and safety and vocational officers.

After consultation with the expert group, the prototype consisted of 7 interactive modules:

1. Pros and cons of disclosure: a list of possible advantages and obstacles was suggested. This module explained that users were to drag and drop the examples that were relevant to them into either the prosor the cons box.
2. Disclosure needs at work: focusing on what employees may need at work to do their job well and stay healthy. This module gave several examples of needs based on the expert’s opinions on accommodations that were typically required by those with mental health conditions at work. Users were asked to select how important the example needsare at work to them on a scale of unimportant to very important.
3. Disclosure values at work: this module provided examples of values that may influence disclosure decision making. Users were provided with 2 opposite options and to slide a scale to the option that best suits their values. An example is “I value… Being open and honest versus keeping private.”
4. When is the best time to disclose: this provided employees advantages and obstacles for each of the following options: in a one-on-one meeting, in a chat at work, in the pub or social event, in my review, after I have a good bond with my supervisor or coworkers or never.
5. Who have they disclosed to in the past: this section provided participants with a space to reflect on their previous experiences by selecting who they may have told, for example, a spouse and the selection of whether the experience was positive or negative.
6. Who is the best person to disclose to: this provides employees with advantages and obstacles to methods of disclosure and/or nondisclosure with the following options: keep it a secret, only tell trusted people, tell anyone, tell everyone, and tell no one.
7. Summary of making the decision (based on the individual options selected in each module) presented to each user.

Each of the first 6 modules allowed space for users to enter their own options for consideration.

We decided to develop a prototype before the participatory design as we wanted to ensure a safe tool was developed. It was first and foremost important to develop the tool based on aspects that have been evaluated and shown to be effective in the previous CORAL paper-based tool. As every country has specific requirements, we developed the content specific for use in Australia.

### Participatory Design Process

The workshops followed the design of Peters et al [19], including individual, whole group, and small group participatory design activities with the main facilitator (ES).

Participatory design was used as it involves users in the design process, focusing on user-centered orientation to draw out user perspectives, preferences, and ideas for the co-design of technologies. Participatory design is increasingly being used as a means of empowering end users by involving them in development [20,21]. Furthermore, participatory methods provide a means to create a democratic and destigmatized space in which to discuss complex topics such as the disclosure of mental health concerns.

These activities included individual reflection and collaborative ideation. The facilitator guided the participants to create ideas for desirable prototype functionality and characteristics. Participants were provided with both interactive and paper versions of the prototype. These consisted of the proposed content of the decision aid tool and its modular design. Participants generated ideas for desirable functionalities and website characteristics and provided feedback on draft screen designs for a prototype. The structure of the groups is provided in Multimedia Appendix 1.

**Figure 1:**
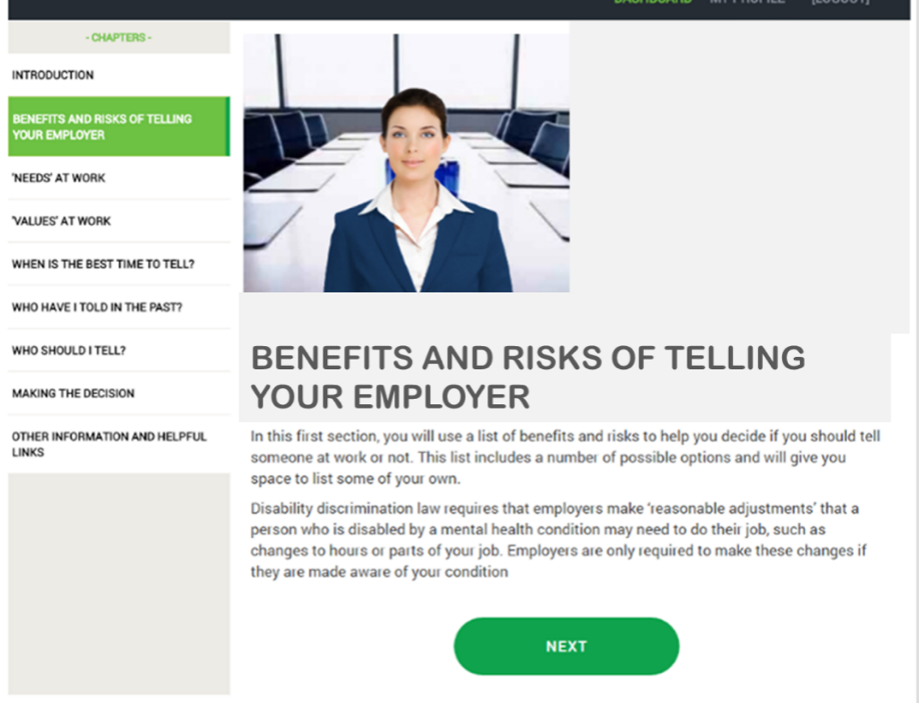
Example of avatar used in workshops as a prototype.

Participants in the groups were also shown a video example of a virtual avatar (an example is shown in Figure 1). This avatar was designed to read sections from within the tool to the participants. Many commercial platforms such as SitePal have been designed for and evaluated within health research and are often used as virtual coaches [22]. We decided to use SitePal because the avatar’s appearance, voice, and even accent were customizable by the developer and allowed users to select their preferred avatar features. This avatar uses the artificial intelligent markup language (AIML) introduced by Wallace [23]. AIML is known to possess *the most human computer*–like features with facial expressions of emotion and nonverbal interaction exhibited by the avatar. Furthermore, it has the ability to handle dialogue flows and closed-ended questions.

### Data Collection

Data were collected through audio recordings and resulting *artifacts* created by participants demonstrating their ideas and comments as well as paper-based forms, sticky notes, and field notes. The workshops were conducted at organizational sites and lasted approximately 90 min.

Semistructured topic guides and activities were designed to assess the process and content of the proposed web-based decision aid tool. The sessions were audio recorded and transcribed verbatim, and the accuracy was verified. The transcriptions were deidentified, and each participant was given a participant identification number as a pseudonym to protect anonymity. Only the lead researcher (ES) had access to the participants’ identification. After the workshops, the facilitator (ES) and principal investigator (NG) met to review the techniques used in facilitation as a debrief. Transcripts were analyzed as soon as possible after each interview. After the 2 groups, participants were corroborating ideas about the design of the tool.

### Data Analysis

Two researchers (the first author and a trained qualitative researcher) independently coded workshop transcriptions using NVivo 11 (QSR International). The investigators used the framework analysis method by Ritchie et al [24] to analyze the data [25]. This method allows for the structured identification of commonalities and differences in qualitative data and helps to draw descriptive and explanatory conclusions clustered around common themes. It is especially useful when multiple researchers are coding the same data set by providing an open, critical, and flexible approach to allow for a rigorous step-by-step qualitative analysis [26]. This method uses 5 stages; the first stage, familiarization, began when investigators started transcribing the data. A thematic framework was then identified by 2 investigators, and each transcript was coded separately. The coding involved an iterative process as the codes were refined, whereas discrepancies were resolved by a third investigator (NG). Next, key themes of direct quotes were indexed to demonstrate the richness of the themes. Subthemes within these key themes provided a more in-depth understanding. In the fourth stage, themes were charted so that the key and subthemes could be easily identified, and data may easily be traced back to its original source.

Thematic analysis focused on categorizing data to inform the development of features and content of their preferred website. Initial coding involved attaching labels to text segments to identify themes related to the research questions, that is, what type of components, including language, style, and content, would avoid potential stigma and ensure elements of clear value for users. The analysis progressed iteratively with independent re-reading of transcripts and re-examining themes against the raw data to further refine the themes and identify subthemes. Discrepancies in coding were regularly discussed and resolved with both coders and with the senior researcher (NG).

## Results

### Participants

The 2 workshops were attended by 13 of the 23 potential participants invited (57% response rate). The majority were from the transport company (9/13, 69%), were male (8/13, 62%), and had full-time employment (11/13, 85%; Table 1). Of the total, 6 employees had previous experience disclosing their own mental health condition (employees: E1-E6), and 7 were in a supervisory role and had previously received disclosures (supervisors: S1-S7). Each workshop contained a mix of individuals from the authority and disclosed groups. With such a small group, we wanted to ensure that the participants were not identifiable, so minimal demographic information was collected. This was an important requirement from both participating workplaces.

**Table 1:** Demographic information for participants.

Category			Values, n (%)
**Worksite**			
	First responder association	4 (31)	
	Transport company	9 (69)	
**Gender**			
	Male	8 (62)	
	Female	5 (38)	
**Employment status**			
	Part time	2 (15)	
	Full time	11 (85)	
**Category**			
	Disclosure	6 (46)	
	Authority	7 (54)	

### Main Themes and Insights

The workshops resulted in 2 overarching themes: content and features.

The *content* theme included the following 4 subthemes:

1. Overall usefulness of the tool.
2. Information on disclosure options.
3. Language preferences, including acceptable mental health terms.
4. Style preferences, including the length of the intervention.

The *features* theme included the following 2 subthemes:

1. Desirable features.
2. Undesirable features.

### Content

#### Overall Usefulness of the Tool

Overall, the participants felt positive about the tool. They found the content and purpose of the tool to be useful and suggested that there would be a benefit for those who were struggling with their mental health but were not sure where to start asking for help:

Self-explanatory, it makes sense.

Employee 1

This would help those struggling.

Employee 3

I thought it was quite good actually. I really thought the questions were good, from my point of view. There’s enough of a broad area covered that they will work.

Employee 4

The positive aspect is that it shows that there are lots of avenues that you can take and just because you’re not going to tell one person doesn’t mean you can’t tell anybody, provided your workplace doesn’t have an issue with confidentiality or obligations. It sends a positive message to them.

Employee 6

#### Information Around Disclosure Options

One of the key themes identified was how the tool should present information on *specific disclosure options* within the workplace. There were 3 modules that were discussed under this theme.

1. “When is the best time to tell?” This provided employees advantages and obstacles for each of the following options: in a one-on-one meeting, in a chat at work, in the pub or social event, in my review, after I have a good bond with my boss or coworkers or never.
2. “Who have you told in the past?” This section provided participants with a space to reflect on their previous experiences.
3. “Who should you tell?” This provided employees advantages and obstacles for methods of disclosure and/or nondisclosure following options: keep it a secret, only tell trusted people, tell anyone, tell everyone, and tell no one.

Both groups had concerns about guidelines in the prototype suggesting disclosing during a *specially arranged meeting*, which was provided as an option in the *when is the best time to tell* module. This was thought to imply that the meeting may not be confidential:

I’d probably lose “specially arranged” but that’s just me or in a meeting, sounds too formal and might not be confidential.

Employee 2

I would change the wording from “formally arranged meeting” to “one-on-one meeting”. Just sounds more confidential.

Employee 3

As a result of this feedback, the phrase *one-on-one meeting* replaced *formal and specially arranged meeting*.

There were some concerns regarding the information guidelines. Some participants expressed concerns that the options suggested for reasonable accommodations might only be suitable for those working a typical 9 to 5 job in an office setting and not applicable to those working shift hours or out-of-office roles:

All needs mentioned seem to fit with those working nine-to-five jobs. I don’t know if these would fit with, for example, shift workers.

Supervisor 5

This doesn’t seem to address people who don’t ever work in the office.

Supervisor 1

Some people might need a complete change in job, not just modifications to their current job.

Employee 4

To address these concerns, some options that would suit nontypical working hours were discussed in the workshop. Some examples of options for the *needs* for adjustments, individuals, roles, and developers implemented were “to be able to meet with my boss and co-workers more often” and “to have more flexible hours.”

#### Language Preferences

Many preferences were expressed for the language to be used in the tool, particularly around words in the prototype that seemed to hold negative connotations. Specifically, both groups reported that the words *disclosure*, *cons*, *risks*, and *disabled* should not be used as they may portray that a mental health condition is *wrong* or *blame worthy*:

Obstacles may hold less of a negative connotation; cons feel like they have done something wrong.

Employee 1

Using the word “disclosure” tends to have a negative connotation it’s like I’ve got to put something on paper, and I’ve got to disclose something bad or wrong. “Reach out”, or “help” might be better.

Employee 1

“Affected” is probably a better word to use than “disabled” as people might not see themselves as disabled.

Employee 2

As a result, the term “obstacles and advantages” was replaced with “pros and cons” and “disclosure” was replaced with “telling or tell.”

The first responder group noted that certain words may have a specific meaning or be more commonly recognized within their industry. For instance, they preferred the word(s) “understanding or empathy” over “sympathy” and “education” over “teaching”:

Some language naturally skews away from, for instance, something like “sympathy about my mental health condition” wouldn’t be used. They don’t want sympathy. I think it should be “understanding about my” maybe “empathy” or “supportive approach to” or “understanding” or “support about my. . . .”

Supervisor 2

Potentially the same with “teaching others is not important” maybe something like “it’s not a priority” or education about mental illness is probably a better word than teaching for the leaders in the organization.

Supervisor 4

These suggestions were implemented into the tool: “sympathy” was replaced with “understanding” or “empathy” and “teaching” was replaced with “education” or “training.”

##### Acceptable Terms Around Mental Ill Health

Several common terms were used to describe mental health conditions by the participants.

The authority group commonly used *mental health space, mentally not feeling well, mental health case,* and *mental health issues.*

The disclosure group used *mental health “basket,” mental state, mental problem, mental health issues, and mental illness.*

There was 1 common term between the 2 groups and that was *mental health issues*. As a result, the tool adapted language to suit the use of this commonly used phrase and was implemented when referring to concerns surrounding poor mental health.

To understand the common slang used for symptoms related to mental health conditions, the coding team noted colloquial words and phrases that were commonly used. The following were used throughout both workshops: *struggling/struggle, not coping, cup is filled up, suffer/suffering, hit the fan, send over the edge, trigger(s)/triggered, strain, stressed, and weak(ness).*

Although there was no appropriate place in the tool for each of the commonly used slang, the following terms were implemented where appropriate: *struggle and stressed.*

A similar approach was used to identify the common words for the clinical services available for addressing mental health conditions and their related symptoms. Participants referred to the following often: *psych services, employee assistance program (EAP), counselling/counselor/psychologist, workers comp, therapy, get support, and support person/group.* The tool implemented a *get support* section in which links to find *psychologist* and *counseling* were added.

Participants made helpful suggestions that within the text for each section of the prototype, it may be important to reassure users that there is no right or wrong answer at any point. This was added to each instruction section for all modules in the tool:

Advise in the starting captions that there’s no right or wrong answers to make sure answers are honest.

Employee 5

#### Style Preferences

##### Length of Intervention

Overall, participants felt that the prototype intervention was the right length, easy to use and understand, and helpful and that it would be of use to those who would be considering disclosure. Participants felt that the tool would educate employees about disclosure options:

It’s a good length and doesn’t take too long

Employee 4

I think this isn’t too long for people who aren’t feeling well, I would’ve liked as much information as possible.

Employee 5

I think it would generally cover every occupation. There’s not too much.

Employee 4

### Features

#### Desirable Website Features

There were several desirable features and characteristics that were requested consistently throughout both groups that were clustered into 4 themes: (1) measuring mental health symptoms, (2) resources, (3) disclosure and industry specificity, and (4) interactive functions.

#### Measuring Mental Health Symptoms

### Mood Scale

Many participants suggested that having somewhere to log their mood before and after using the tool would be a great way to see how they were feeling as a *check in*:

I would like to see a scale of mood to measure at the start to see how I’m feeling when I am ready to disclose.

Employee 4

Something to measure “what has brought you here today”, I am feeling down this that.

Supervisor 2

### Self-Assessment of Symptom Tool

There was strong interest in a mental health assessment tool that measures the current mental health state; the idea was to see if this may provide further knowledge of individual symptoms and help those who have not ever received a diagnosis to understand or validate their symptoms:

Needs an assessment scale at the start before it’s used.

Employee 4

An assessment tool would be useful to see if what I’m feeling is really a mental illness or not.

Employee 5

### Feedback of the Current Mental Health Score

A couple of participants felt that after the self-assessment, it may be beneficial for participants to receive feedback about their current mental health state. The idea was that this information could be provided via an email or a pop up. This did not receive any disagreements in either workshop:

I think its missing diagnose section. I need to know results from some symptoms if I’m struggling to see if it’s really a problem.

Employee 4

#### Resources for Providing Mental Health Support

### Option to Send Feedback to a Clinician

Some participants requested a function where feedback could be sent to their own psychologist or possibly even the EAP. One participant suggested a print option so that they could take the results along to a future meeting around disclosure:

It would be good to be able to share the results with support people to make sure you’re getting the right help or print and give it to someone when you’re disclosing as a backup.

Supervisor 7

### Easy Access to Urgent Help via Pop-Up or Contacts Page

Most participants suggested and/or agreed that links to mental health support organizations both within the workplace and externally were a must, particularly as the website may be accessed outside of work hours and some may be unwell or even suicidal. This was met with strong support from the authority group:

Add a section on having a contact person that you know you can get help.

Supervisor 4

A section on either inside their workplace or like beyondblue or something would be good to have to show people who they should contact to where they should go if they aren’t feeling great.

Supervisor 3

Add a section on where to get assistance.

Supervisor 3

### Option to Request Contact From an External Provider

The idea that someone from outside of the workplace should monitor participants’ responses and contact them or that participants might have an option to ask for help from a member of the research team was suggested to make sure participants are being helped:

I would like to see a ‘would you like to be contacted’ section mainly for those who score high on mental health because they probably won’t get help.

Employee 3

Add a section on having a contact person that can contact and give you help.

Employee 1

#### Characteristics: Disclosure and Industry Specificity

### Tailoring for Industry Specificity

The idea of the intervention being tailored to suit each industry was prevalent in both workshops. This idea was repeatedly requested by a majority of participants based on the idea that industries would require specific needs that may not be translatable across all industries:

I think it should be based on each industry, as our needs would be different to another organization.

Employee 2

The needs suggested should be workplace specific as not every job has the same needs.

Supervisor 2

#### Interactive Functions

### Notifications With Positive Daily Messages or Emails

Participants suggested that sending emails or messages with positive facts or mental health statistics may help participants feel like they have ongoing support:

Ongoing help and reminders will make it feel more supportive and helpful.

Supervisor 3

It wouldn’t be a bad idea to have push notifications with positive messages or mental health stats.

Employee 6

If they (messages) were short messages people would benefit.

Employee 5

### “Get Help” Chat Section to Interactively Chat With Someone External

The idea that giving participants someone to interactively chat with was mentioned in both workshops. Participants felt a chat function would allow them to feel like they have ongoing support:

At the moment the extent of the resources is go onto the intranet and find the resources and download the PDF and it all feels a bit static that’s why a chat type thing would help with getting support or feeling supported.

Supervisor 2

### Anonymous Community Forum to Share and View Stories of Lived Experiences of Disclosure

The idea of having an anonymous community forum for individuals to share stories had mixed responses. Some participants felt this would be a useful function as long as it was anonymous, not identifiable, and monitored to avoid cases of bullying:

As long as a community forum is not identifiable it would be a good tool.

Supervisor 2

I think information about it [mental illness] and sharing stories with each other is a good idea, useful.

Employee 4

The team might to vet who’s on there as there’s a risk if you’re talking to some people who are also depressed it might make people worse.

Employee 3

### Testimonial Section From Others Who Have Disclosed in the Past

Participants felt as though a testimonial section about the tool would encourage other employees to use the tool, especially if the testimonial was positive and shared a positive outcome:

*People love hearing about other people’s successes so if someone comes back and says, I did the tool and I spoke to my boss and my workplace and everythingwent well*. [Employee 5]

### Videos or Scripts of Example Discussion or Guidance About How to Disclose

One participant suggested that giving participants access to scripts or examples of disclosures in short videos may help those disclosing to feel less isolated and more informed:

*I think information is power and that kind of thing, facts about disclosing might make people feel like they aren’tisolated*. [Supervisor 1]

### Disclosure Decision Making

There was strong interest in having the tool provide a *score* or give recommendations. Participants seemed to want to be told what to do in terms of choosing disclosure or nondisclosure. For instance, the tool could advise on which disclosure option to choose based on the answers selected by the participant. Other suggestions proposed that the answers could be summarized at the end of the tool:

It would be helpful to have a score or suggestion page to tell people which option is best based on what they have said.

Employee 1

### Undesirable Website Features

Participants in the groups were shown the video example of a virtual avatar (an example is shown in Figure 1). Participants were advised that the avatar and the background could be customized to their liking.

In both groups, a majority of the participants reacted unfavorably to the use of an avatar. In total, 11 participants reported that their main concern was that the cartoon figure may be interpreted negatively, suggesting that the tool may not be taking the condition seriously enough. Another participant suggested that in remote areas, it may become frustrating if the avatar was pausing because of poor internet connection:

May be too light-hearted for such a serious problem.

Employee 5

May make people feel like they’re not being taken seriously with a cartoon.

Employee 6

may be frustrating if you’re in a remote area with no Wi-Fi or not a strong internet connection.

Employee 6

Probably better with the younger crowd you know that interaction if you’re given something to read it might just go over their head.

Employee 1

Feel like it’s making jokes about mental health or makes it seem child-like.

Employee 2

Participants were asked to describe the avatar in a few words, all of which were negative, for example, *creepy, childlike, annoying, not professional,* and *not confidential.*

**Figure 2:**
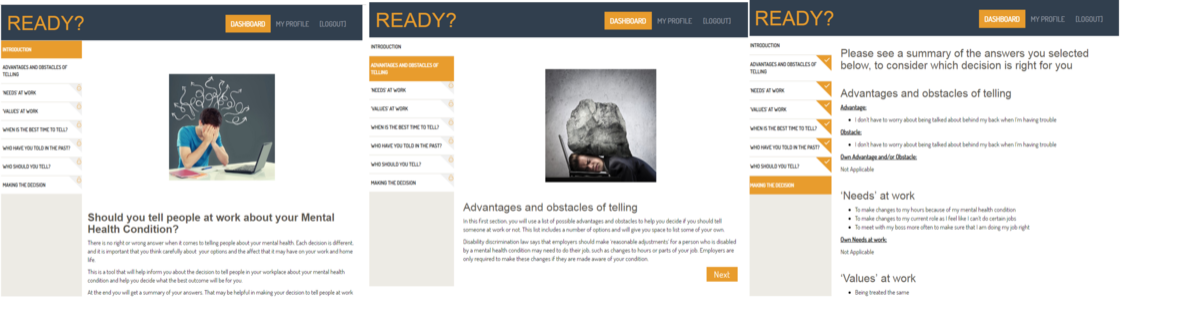
Example of the adapted READY intervention dashboard.

The final 7 modules discussed in the prototype were approved by the participants as: advantages and obstacles, *needs* at work, *values* at work, when is the best time to tell, who have you told in the past, who should you tell, and making the decision. A visual example of the final version of the tool is provided in Figure 2.

## Discussion

Digital and mobile health interventions have become more popular in workplaces because of their accessibility, cost-effectiveness, and confidentiality [16]. The use of digitally delivered decision aids has shown positive results in helping patients make treatment-related decisions [12]. Furthermore, an evaluation of a paper-based decision aid tool for employees showed promising results in assisting in making decisions about mental ill health disclosure options [14]. However, a primary reason why digitally delivered interventions can be poorly implemented is that a tool can only be effective if it engages its intended audience [18]. When developing interventions, developers should consider the end user’s needs and preferences. A key aspect of ensuring that the tool is effective is a collaborative co-design design approach.

This study used a participatory design approach to allow all stakeholders, including developers, researchers, experts, and end users, to collaborate in co-designing the tool. This approach was used to ensure that READY, the decision aid tool, was more likely to be engaging and effective for its intended audience. We share our key findings including (1) aspects of the tool where all stakeholders were in agreement, (2) aspects where some stakeholders disagreed and adaptations were implemented, (3) where disagreements could not be implemented because of financial constraints, and (4) where there was misalignment between stakeholders and how to decide on a balance, for instance, how the developers came to decisions about which stakeholders’ ideals were implemented and why.

### Areas of the Prototype Where All Stakeholders Were in Agreement

Overall, participants felt positively about the prototype put forward. They found the content and purpose of the tool to be useful and all modules necessary. Participants suggested that there would be a benefit for those who were struggling with their mental health but were unsure where to start asking for help. Interestingly, many changes were superficial features such as preferred language, which only required minimal adaptations.

Although participants noted that the tool contained a large amount of written text, they did not consider this something that required changing as they acknowledged that disclosure is a complex area and requires thorough information.

### Adaptations That Were Integrated Into the Tool Because of Disagreements With the Prototype

Several changes were made to the language of the tool, for example, *pros and cons* and *risks and benefits* were changed to *obstacles and advantages* to allow for optimal acceptability of the tool. It was important to both groups that the word *disclosure* was not used. This word seemed to carry negative connotations, thus, developers referred to disclosure in the final version as *tell*.

As the intended end users were from Australia, we developed the language in the tool for Australian-specific industries. Interestingly, participants felt that the language should be *industry specific* as common colloquialisms are used across different industries. For international adaptations of the tool, future research may consider whether the language differs within industries across countries; for example, the slang used in male-dominated industries in Australia may differ from that in the United States.

Several desirable interactive features were added to the final version of the tool, including the addition of mental health feedback scores. The participants were provided with an email regarding their depression and stress levels. This was deemed important as, in our previous qualitative analysis, it was identified that employees who did not have a formal diagnosis had little knowledge of whether their mental health symptoms would meet diagnostic criteria [9].

It was decided that mental health support resources were an important feature of the tool. Mental health resources were added throughout the website. Participants could click on the *get support* tab to obtain phone numbers and links to relevant mental health resources in their area. If participants indicated significant depression or suicidality when answering the mental health screening questions, they were automatically provided with these links and phone numbers and were advised to contact a general practitioner for more information on their mental health.

### Desirable Features That Could Not Be Integrated Because of Financial Constraints

The participants suggested some interesting features that could not be included in the scope and budget of this tool that warrant focus in future developments. First, an anonymous community forum provides helpful or positive stories. The research team agreed that it would be important to share success stories around disclosure as all too often we hear about negative injustices and discrimination that occurs as a result of disclosure. The Chief Executive Officer of the lead mental health charity in Australia herself suggested in a national newspaper that employees should not disclose their mental health conditions, “Don’t [disclose], because you might not get that promotion, you might get the sack, there might be repercussions” [27]. However, studies suggest that those who decide to disclose more often than not have positive experiences [28]. Future versions could benefit from a monitored anonymous community forum where other employees may share their success stories or helpful advice. This development team did not have the ability to monitor a community forum under the scope of this project. Second, the suggestion was made about video testimonials again sharing success stories or helpful advice on how and when to disclose. As a result of budget, staffing, and time constraints, the developers were unable to provide this feature; however, it is strongly recommended in future versions as this would be a means of addressing the negative reporting bias.

### Areas of Misalignment Between End Users and the Developers, Expert Groups, and Researchers

There were 2 main areas where misalignment appeared between the workers and organizational preferences and the prototype developed by experts. The first was the suggested use of an avatar by the developers. Avatars have previously been used in medical settings. The developers were initially interested in including an avatar into the intervention to assist those with lower literacy levels. Previous studies utilizing avatars to deliver medical information to patients with low literacy indicated that the avatar provided an additional authoritative source for their medical information and a majority preferred receiving the information via the avatar compared with reading the information themselves [29]. An avatar assisted veterans with postdeployment distress in help-seeking decisions. Those randomized to an avatar group exhibited significantly greater likelihood of recognizing their symptoms and seeking help for their mental health concerns compared with a control [30]. However, in this scenario, with such a strong negative response from the worker preference discussions, it was decided not to include the avatar to minimize potential harm as participants suggested that the use of a *cartoon* may be downplaying the seriousness of mental ill health.

The second area of misalignment was the provision of a final score or recommendation on disclosure by the tool. Although workers and some of the expert group wanted a final score of whether or not users should disclose, the developers felt there was no fair or safe manner to provide either a score or a recommendation for several reasons: (1) decision aid tools are not designed to make decisions for the users but aim to increase autonomy and self-determination; (2) it is difficult to ascertain the relative weight of each component as it is unknown what value to place on each selected response. For instance, if 5 small positive reasons for disclosing outweigh one very negative reason, what relative value would “I will get sacked” have compared with “I will feel honest”; (3) a recommendation led to unforeseen consequences. If the tool suggested that the employee should disclose and, as a result of the said disclosure, the employee is fired from their position, there are potential governance and medicolegal consequences that might vary enormously by the employer and occupation, and (4) the tool can only collect information requested. The tool may miss key aspects in certain scenarios, for instance, mandatory reporting requirements in health care.

As an alternative, the final intervention included a summary page on which each of the answers selected were summarized on 1 screen such that participants could see the full picture of their selected values, needs, and facilitators to their disclosure or nondisclosure.

### Strengths and Limitations

The participatory design approach supported employees in openly and voluntarily discussing their experience with mental health disclosure. Consistent with Peters et al [19], when participants are given a safe space to discuss mental health disclosures, typically a taboo subject in an occupational setting, they can be highly generative of ideas. Interestingly, the mental health disclosure experiences of employees were discussed openly in the same workshop as the authority group within the same organization, despite these employees reporting only negative experiences and barriers to disclosure [9]. This suggests that employees have an interest in contributing to and improving future disclosure decision making, be it their own or other employees, above and beyond any potential stigmatizing attitudes.

This study was not without limitations. It should be noted that it is likely to have been influenced by a self-selection bias as those who volunteered to participate are likely to represent those more willing to discuss mental health issues. More importantly, the research team only had information to invite participants from the organizations that had disclosed or been disclosed to. Our study did not include anyone considering disclosure or those who were yet to disclose who would most benefit from the use of this tool.

The study was limited by the involvement of only 2 organizations. Therefore, the participant sample was limited in terms of industries represented and geographical location as all participants were from Australia and from male-dominated industries. As such, responses may not be generalizable to other occupational populations.

### Conclusions

In any co-design development, there are certain trade-offs that need to be made between the views of experts, developers, and end users and the available budget [31]. These trade-offs should be carefully considered, although it is important for the uptake, engagement, and usability of the target audience to include input from end users [32]. In this specific instance of a very delicate, personal decision, the end users provided valuable design insight into key areas such as language and were very antipathetic to a key feature, the avatar, which was thought to be desirable by experts and developers. Conversely, the end users only know what they know and they may not be aware of the implications of what they may wish for [33]. In this study, the suggestion of the tool providing a score at the end or advocating disclosure or nondisclosure may have legal ramifications. The co-design with lived experience approach is useful for contributing much to the design, language, and features. The key in this study was balancing the needs of the workers and the potential impacts for the managers and organizations when ensuring legislation and regulation requirements were upheld.

## Appendix

Multimedia Appendix 1 Workshop plan and design.

## Abbreviations

AIML: artificial intelligent markup language
CORAL: Conceal or ReveAL
EAP: employee assistance program
READY: Reducing dEcisionAl conflict, a Decision aid tool for emploYees
