# Supplementary material for: Co-Designing a Web-Based Decision Aid Tool for Employees Disclosure of Mental Health Conditions: A Participatory Study Design Using Employee and Organizational Preferences
Source: JMIR Form Res. 2020 Nov 6;4(11):e23337. doi: 10.2196/23337 (PMC7679208; doi:10.2196/23337)
Supplement: Multimedia Appendix 1 [file formative_v4i11e23337_app1.docx]

Supplementary Material. Workshop plan and design

Psychological context (Personas & Mental Models)

- Attitudes towards openness to mental health
- Attitudes towards disclosing mental health

Language & UI (visual and textual)

- Language they use around disclosure/mental health;
- What is a meaningful way to feedback information to them about disclosure
- How to communicate disclosure in a language that they will appreciate.
- Preferences for conceptualisation of a disclosure decision aid tool
- Preferences for the look, feel, length, content and attitude of the tool
- Preferences for the name of the tool

Context (social, physical, environmental)

- Relationship with others at their own workplace
- A list of people they would turn to for support within and outside their organisation. Why they consider that person a good shoulder to lean on. Any anecdotes of this happening that they are comfortable sharing.

Technical and Interaction

- Preferred features, (eg. Interactive audio features or use or avatar)
- Preferences for tool communication (eg. text message/reminders.)”

| **Activity/Method** | **Min** | **Description** | **Materials** | **Outcomes** |
| --- | --- | --- | --- | --- |
| **Consent** | 20 | 1. Individual consent (depending on the size of the group may take longer) |  |  |
| **Opener** | 10 | 1. Ask everyone to say their name/icebreaker. 2. Summary of why we’re here, their expertise, and what they’ll be co-designing/contributing to. | Flipchart, markers, stickies |  |
| **Discussion on disclosure** | 10 | Participants are asked to initially comment on previous experience of disclosure or using a tool to help them decide | Flipchart, markers, post-it notes, pens | - How people think about disclosure, - Attitudes towards current tools - What do they take into account when disclosing - What things do they consider changing? - Language they use around disclosure/mental health; - How to communicate disclosure in a language that they will appreciate. |
| **Present the content of this tool** | 20 | In small groups, they will be asked to use sticky notes to make comments on the content of the tool with certain questions in mind (10min) Each group presents results. (5min) (not relevant if interviewing one-on-one, time will still be given but not in groups) | Include a list of questions to focus on, flipchart, markers, post-it notes, pens | - Preferred features - Preferences for tools communication (eg. Interactive audio features or use or avatar) - Language they use around disclosure/mental health; - How to communicate disclosure/mental illness in a language that they will appreciate. - Preferences for the look, feel, length, content and attitude of the tool |
| **“Design the tool”** | 20 | In small groups, participants draw/write what features the tool should remove, merge, add to the tool to show how it would work and what features it should have. (10min) Groups present findings (5min) (not relevant if interviewing one-on-one, time will still be given but not in groups) | Include a list of questions to focus on, flipchart, markers, post-it notes, pens | - Preferred features - Preferences for disclosure feedback communication (eg. text message, reminders, should there be a score or personalized feedback.) - Language they use around disclosure/mental health; |
| **Tips for the designers – closing** | 10 | Ask participants what advice they would give to the designers and developers of this tool and any other thoughts. Collate onto board. |  |  |
| **Total Duration (hours)** | 90 mins  may vary depending on the size of the group |  |  |  |
